# Machine Learning-Based Prediction of Bioprinting Quality Using Integrated Experimental and Computational Data

**DOI:** 10.3390/bioengineering13101148

**Published:** 2026-10-01

**Authors:** Khulood Abu Maria, Sara Abu Tarboosh, Adi El-Dalahmeh, Ola Tarawneh

**Affiliations:** 1 Department of Computer Science, Al-Zaytoonah University of Jordan, Amman 11733, Jordan; 2 Faculty of Pharmacy, Al-Zaytoonah University of Jordan, Amman 11733, Jordan; sara31122000@yahoo.com (S.A.T.); ola.tarawneh@zuj.edu.jo (O.T.); 3 Department of Cybersecurity, Al-Zaytoonah University of Jordan, Amman 11733, Jordan; a.eldalahmeh@zuj.edu.jo

**Keywords:** 3D bioprinting, machine learning, extrusion printability, alginate–methylcellulose, drug delivery, computational fluid dynamics, stacked ensemble

## Abstract

Three-dimensional (3D) bioprinting of hydrogel inks is an enabling technology for tissue engineering and the fabrication of personalized drug-delivery systems. However, in most laboratories the extrusion parameters are still optimized by slow, resource-intensive trial-and-error, because printability depends on the combined and often nonlinear influence of ink composition, extrusion pressure, and nozzle geometry rather than on any single variable. Alginate–methylcellulose (Alg–MC) is a widely used pharmaceutical bioink whose shear-thinning behavior makes this optimization particularly demanding. Here we developed a physics-informed machine-learning framework that predicts extrusion print quality before printing. A hybrid dataset of 3280 labeled samples was assembled by combining laboratory extrusion experiments, computational fluid dynamics (CFD) simulations of nozzle flow, and controlled augmentation with a deep belief network (DBN). Five physics-informed features encoding pressure-to-diameter and shear-rate interactions were engineered to embed rheological domain knowledge. Five base classifiers alongside two ensemble strategies, seven models in all, were benchmarked under five-fold stratified cross-validation. The voting ensemble performed best (accuracy, 93.3%; F1 = 0.929), narrowly ahead of the stacking ensemble (93.3% accuracy; F1 = 0.928). Alg:MC ratio, extrusion pressure, and the pressure–diameter interaction term stood out as the dominant predictors, consistent with established alginate rheology. On external validation—withholding one composition ratio and one needle gauge entirely from training and testing on those genuinely unseen conditions—accuracy was 75.9%, a stricter test than a random held-out split. Overall, the framework offers a practical, interpretable way to cut down experimental burden in pharmaceutical bioink development.

## 1. Introduction

Three-dimensional (3D) bioprinting has become an important tool in tissue engineering and in the manufacture of personalized, drug-loaded dosage forms, owing to printer hardware that gives precise control over temperature, extrusion pressure, ink selection, and nozzle inner diameter [1,2]. For hydrogel-based inks, successful printing requires careful matching of pressure to the rheological properties of the shear-thinning bioink, and the extruded filament must retain its intended geometry after leaving the nozzle [3,4,5]. Because small changes in pressure or nozzle diameter can shift the ink between stable and defective extrusion regimes [4,5,6,7], laboratories typically rely on repeated trial-and-error, which wastes material and limits systematic exploration of the parameter space [6,7,8]. Conventional printability indices provide only partial guidance because they evaluate each variable in isolation and do not capture the interactions between them [7,9,10].

Machine learning (ML) has recently been applied across the pharmaceutical 3D-printing pipeline to predict printability, filament properties, and drug release, and to reduce the number of formulation experiments required [11,12,13,14,15,16,17,18,19,20,21]. Notably, Elbadawi and co-workers demonstrated that ML models trained on hundreds of formulations can predict the printability and fabrication parameters of 3D-printed medicines with high accuracy [18,19], and related work has extended this approach to over nine hundred drug-delivery systems and to selective-laser-sintered products [20,21]. However, most published models are trained on relatively small, single-system datasets and operate as black boxes whose predictions are difficult to relate to the underlying extrusion physics [22,23,24]. This limits their transferability to new inks and printer configurations and motivates approaches that combine data-driven learning with physical domain knowledge.

We address that gap here for alginate–methylcellulose (Alg–MC), a pharmaceutical hydrogel bioink, with a physics-informed ML framework that predicts extrusion quality before printing. It combines experimental prints with CFD simulations of nozzle flow and with engineered features derived from rheological principles, so that pressure and nozzle geometry explicitly shape the model’s representation of the internal shear-rate field. We also quantify how the polymer ratio influences filament stability and compare model performance with and without the physics-informed features.

This study makes three contributions. First, a structured hybrid dataset (experimental, CFD, and DBN-augmented) that cuts down the number of physical screening experiments needed. Second, pre-print quality prediction directly from process settings, without the imaging or rheometric measurements needed at inference time by camera-based systems. Third, model behavior that can be tied back to verifiable physical principles, which improve interpretability. We show that a stacking ensemble trained on this dataset predicts extrusion quality accurately and recovers the expected physical dependencies along the way, giving a practical decision-support tool for bioink optimization in resource-limited laboratories.

## 2. Related Work

Research on ML for bioprinting and pharmaceutical 3D printing falls broadly into three directions. The first is real-time extrusion monitoring: Kelly et al. [15] combined an under-nozzle camera with convolutional neural networks trained on real and synthetic images to catch and correct under- and over-extrusion within seconds. Closed-loop systems like this work well for post-deposition correction, but they cannot anticipate defects before extrusion starts and cannot see the flow inside the nozzle itself. The present framework takes a different approach, predicting quality before printing from physical process inputs rather than surface images.

The second direction is parameter optimization: Shin et al. [16] studied how pressure and nozzle dimensions affect droplet formation and filament structure, while Sheikh et al. [17] used CNNs to predict spheroid viability, though accuracy dropped for geometries outside the training distribution. Both studies point to a recurring limitation—models tend to inherit the narrow scope of their training data—which the present work addresses by training across a wider parameter space and using physics-informed features grounded in shear-thinning flow behavior.

The third direction is predictive modeling of ink behavior prior to printing. In the pharmaceutical domain, Elbadawi et al. developed M3DISEEN, an ML tool that predicts the printability and processing parameters of drug-loaded formulations [18], and showed that rheological data alone can predict tablet printability and drug dissolution [19]; Muñiz Castro et al. later scaled this approach to more than nine hundred drug-delivery systems [20]. In the bioprinting domain, related studies applied ML to biopolymer selection [22] and scaffold-quality prediction [25], but generally relied on limited experimental datasets [23,26] or aggregated heterogeneous data with inconsistent labeling [24]. Machine learning has also proven useful closer to home, in adjacent institutional work: AI-readiness assessments among health-profession researchers and students [27], and predictive modelling applied to institutional and academic data [28]. The same ensembling logic shows up well beyond bioprinting, too—multi-classifier comparison for physiological signal classification [29], super-learner ensembling for non-destructive quality assessment [30], and ensemble deep networks for biomedical diagnosis [31] all report the same pattern: ensemble methods outperform individual learners on structured, multi-parameter classification tasks. Our approach differs in unifying experimental, CFD, and DBN-augmented data into one consistent dataset and embedding physics-derived features, which yields pre-print predictions without any imaging needed at inference time.

## 3. Methods

### 3.1. Workflow Overview

**Figure 1:**
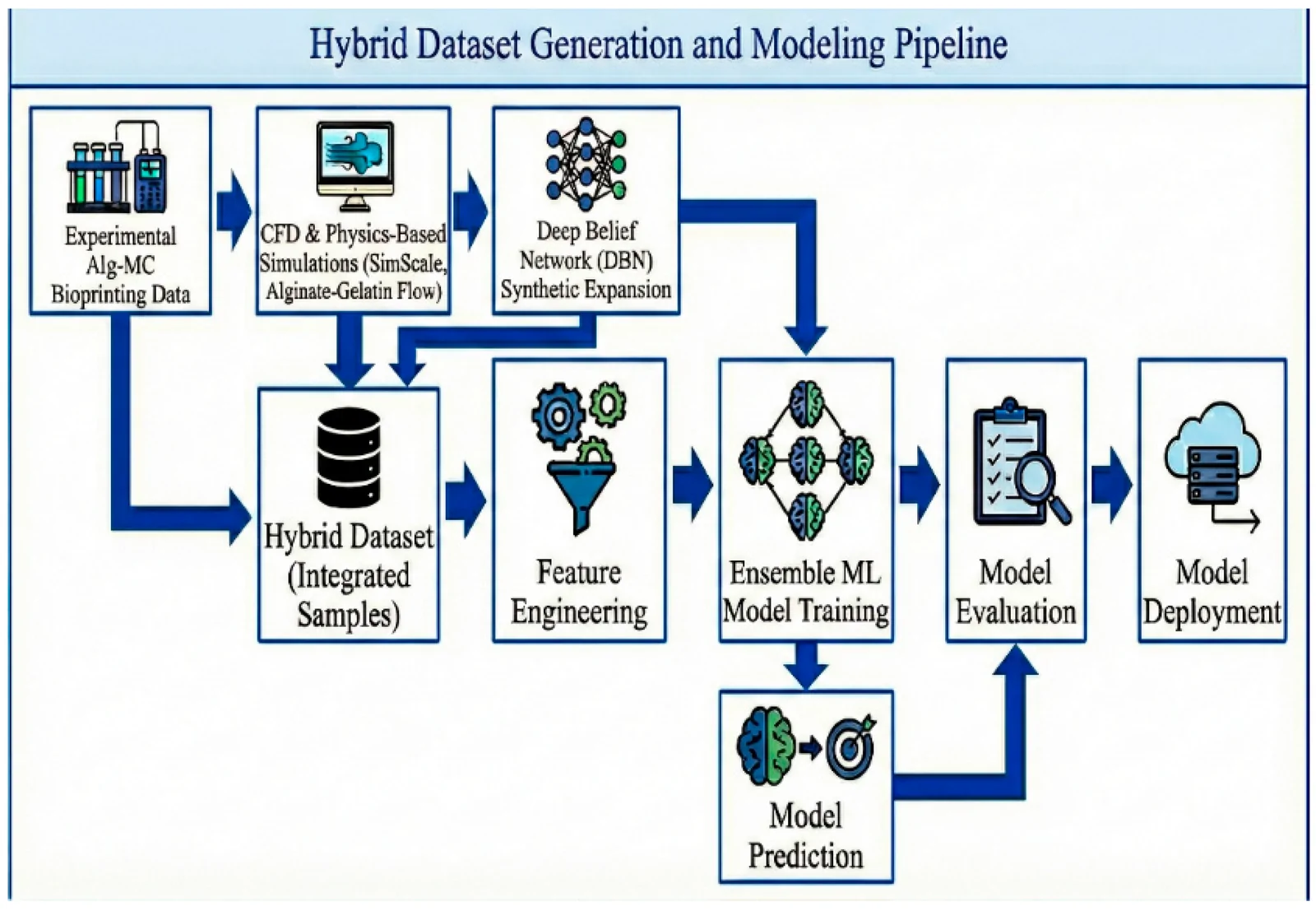
Study workflow. Experimental Alg–MC extrusion data and CFD nozzle-flow simulations are combined and expanded with a deep belief network into 3280 labeled samples, then carried through preprocessing, physics-informed feature engineering, ensemble model training, evaluation, and deployment.

The framework comprises four stages: experimental data collection, CFD simulation of nozzle flow, DBN-based dataset augmentation, and ML training and evaluation. The full pipeline is shown in Figure 1.

The first data source was experimental extrusion testing across defined pressure levels, nozzle configurations, and material compositions. The complete list of input variables, with their ranges and units, is given in Table 1. Of the 3280 samples in the final dataset, 200 (6.1%) came from direct laboratory extrusion measurements. CFD simulation across a systematic sweep of the composition, gauge, and pressure ranges in Table 1 supplied a further 2600 samples (79.3%), and DBN-based augmentation contributed the remaining 480 (14.6%), targeting sparse, under-represented regions of the parameter space as described in Section 3.3 and Section 3.4. The composition grid spans five Alg:MC ratios (1:1, 1:1.5, 1:2, 1:2.5, and 1:3) rather than three coarser levels, and the needle-gauge range runs from 14 to 32 rather than a narrower 18–27 band. This finer, wider parameter coverage is a genuine strength of the dataset, not an incidental detail: it lets the model learn how the printability boundary shifts across intermediate compositions, instead of inferring that shift from only a few widely spaced points. The wider gauge range also means that the reported accuracy already reflects performance across a broader range of practically relevant nozzle sizes than a narrower sweep would have captured.

CFD simulations reached into pressure and geometry regions that experiments alone could not access, and the DBN filled in sparse regions of the parameter space while staying within the physical limits set out in Table 1. CFD simulation supplements experimental data rather than standing in as a separate, independently analyzed dataset: CFD-derived and experimental samples share a single combined parameter space through preprocessing, feature engineering, and model training. Preprocessing itself standardized units, encoded categorical variables, and removed inconsistent records before physics-informed features were added and the ML models were trained and evaluated using accuracy, class-level metrics, and diagnostic plots.

### 3.2. Experimental Data and Printability Labels

We prepared Alg–MC inks in-house, applying identical mixing to every batch to keep batch-to-batch variability down. Composition and process settings were varied systematically within the ranges in Table 1, and we recorded pressure, nozzle dimensions, and Alg:MC ratio for each run. Printability was screened first by depositing a single filament or short line, before moving on to complete structures.

We assigned print quality to three categories—Excellent, Intermediate, and Poor—based on filament cross-sectional shape, line stability over a 30 mm deposit, and visible surface defects. Two trained evaluators assessed every sample independently, and a third assessor resolved disagreements by consensus; inter-rater agreement was strong (Cohen’s κ = 0.83). After labeling, stratified sampling was applied so that no single class dominated the training data.

### 3.3. CFD Simulation of Extrusion Flow

CFD simulations were performed in SimScale [32] for printing conditions that could not be exhaustively tested experimentally. Nozzle flow was treated as steady and incompressible, and the Navier–Stokes equations were solved with a power-law viscosity model (consistency index k = 12.4 Pa·s*n*; flow index n = 0.41) representing the shear-thinning behavior of Alg–MC inks, using values consistent with published rheology for comparable systems [33,34,35,36].

The nozzle mesh was refined in the constriction, where velocity and shear gradients are largest, and mesh independence was confirmed when successive refinement changed the predicted flow rate by less than 2%. A fixed inlet pressure and a zero-gauge outlet pressure were imposed, and simulations were run until residuals fell below 1 × 10^−5^. Die swell, gravitational sag, and post-extrusion viscoelastic relaxation were not modeled and are discussed as limitations in Section 3.9. By design, the CFD-simulated conditions complement rather than duplicate the experimentally tested parameter space, covering combinations—higher pressures, extreme gauge/ratio pairings—that would be impractical to test exhaustively by hand. CFD outputs are simulated flow fields rather than physical filaments, so the visual inspection used for experimental specimens (Section 3.2) does not apply here. CFD-generated specimens were instead labeled with a calibrated proxy criterion: the predicted extrusion flow regime (stable, transitional, or unstable, based on simulated pressure–shear behavior) was mapped onto the same three-class scheme, with the proxy’s decision boundaries calibrated against the flow regimes observed at matched conditions in the experimental subset.

### 3.4. DBN-Based Dataset Expansion

A deep belief network (DBN) was used to populate sparse regions of the combined experimental–CFD dataset [37]. The aim was to generate physically plausible samples in under-represented regions rather than to reshape the existing distribution. The network stacked three restricted Boltzmann machines (128, 64, and 32 hidden units; sigmoid activations) and was trained by contrastive divergence with a batch size of 64 and weight-decay regularization over 80 epochs.

Only synthetic points with low reconstruction error were retained. Two-sample Kolmogorov–Smirnov tests confirmed that every feature’s marginal distribution was statistically indistinguishable between the synthetic and real subsets (all *p* > 0.05), supporting the validity of the augmented data.

### 3.5. Preprocessing and Feature Engineering

Before training, all sources were harmonized: composition ratios became continuous values, categorical fields were one-hot encoded, and records with missing or inconsistent entries were dropped. Features were then standardized to zero mean and unit variance, using statistics estimated on the training partition only so no information leaked from validation data.

**Figure 2:**
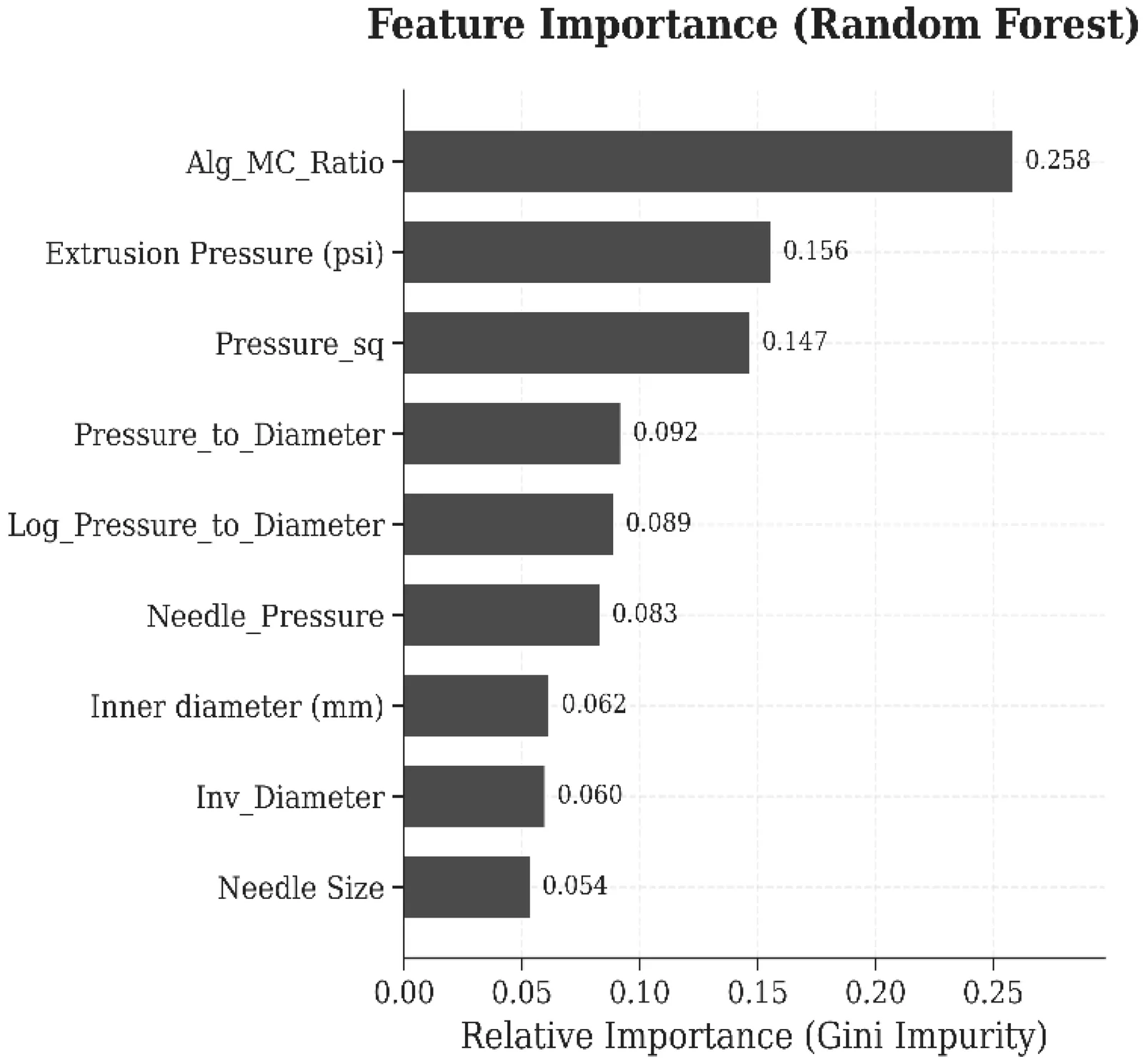
Feature-importance ranking from the random forest model (Gini impurity reduction). P × d (0.330) is the single most influential predictor, ahead of the composition-weighted term (0.274) and log(P) (0.194). Physics-informed features together account for most of the total importance, and P × d—the term most directly grounded in Hagen–Poiseuille shear-stress scaling—contributes more than any other individual feature.

We constructed five physics-informed features: (i) the pressure-to-inner-diameter ratio (P/d), a proxy for nozzle wall shear rate; (ii) a pressure–diameter interaction term (P × d); (iii) the logarithm of extrusion pressure (log P); (iv) the squared inner diameter (d^2^); and (v) a composition-weighted shear sensitivity index derived from the Alg:MC ratio. Figure 2 and the ablation study (Section 3.8) examine their influence. P denotes extrusion pressure (kPa) and d nozzle inner diameter (mm), both defined and ranged in Table 1; r is the Alg:MC composition ratio, expressed as a continuous value. Formally, f_1_ = P/d, f_2_ = P × d, f_3_ = log(P), f_4_ = d^2^, and f_5_ = w·τ(r), where τ(r) is a power-law function of composition ratio and w a fitted scaling constant, both defined below in Section 3.5.

### 3.6. Model Training and Ensemble Structure

Five base classifiers—random forest (RF), gradient boosting (GB), support vector machine (SVM), logistic regression (LR), and k-nearest neighbors (KNN)—were trained under five-fold stratified cross-validation with grid-search hyperparameter tuning. Cross-validation folds were fixed before DBN augmentation so that synthetic points derived from a training fold never entered the corresponding validation fold, preventing leakage. Two ensembles were also built: Cross-validation folds were defined first, using only the real experimental and CFD-simulated samples. A separate DBN was then trained independently within each fold, on that fold’s training partition alone, and its synthetic output was added only to that same partition—no single DBN was ever fit to the pooled dataset after fold assignment. Validation data for a given fold therefore could not influence, directly or indirectly, the synthetic samples used to train the model evaluated on that fold.

- Voting ensemble: majority vote across the five base learners.
- Stacking ensemble: a regularized logistic-regression meta-learner trained on out-of-fold base-learner predictions, with base models held fixed during meta-learner training.

Performance for all models is reported in Table 2.

### 3.7. Evaluation Metrics and Diagnostic Plots

**Figure 3:**
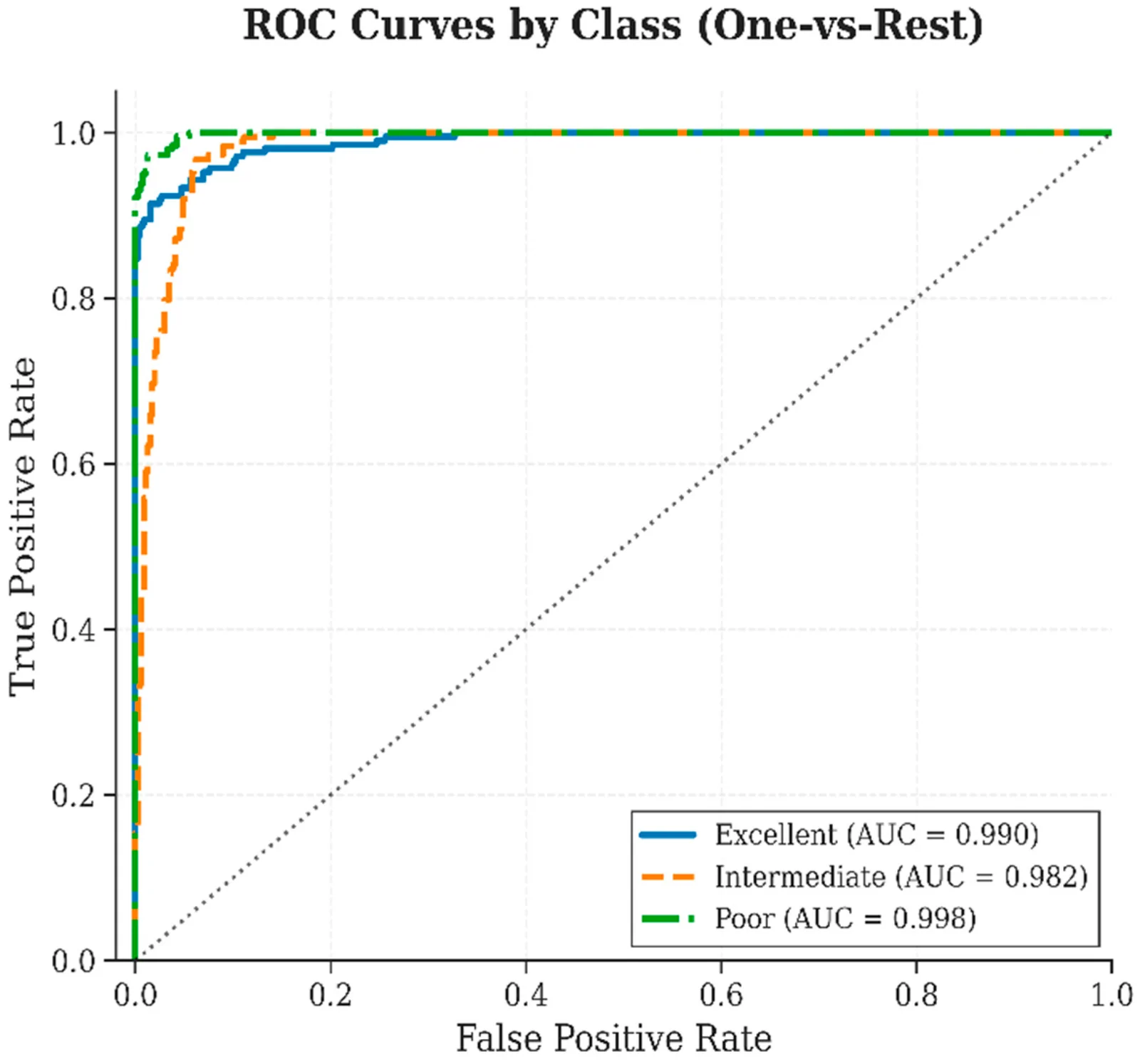
One-vs-rest ROC curves for each printability class. AUC values: Poor = 0.998; Excellent = 0.990; Intermediate = 0.982. All three classes separate well; the Intermediate class shows the broadest uncertainty region, fitting its role as a transition state.

**Figure 4:**
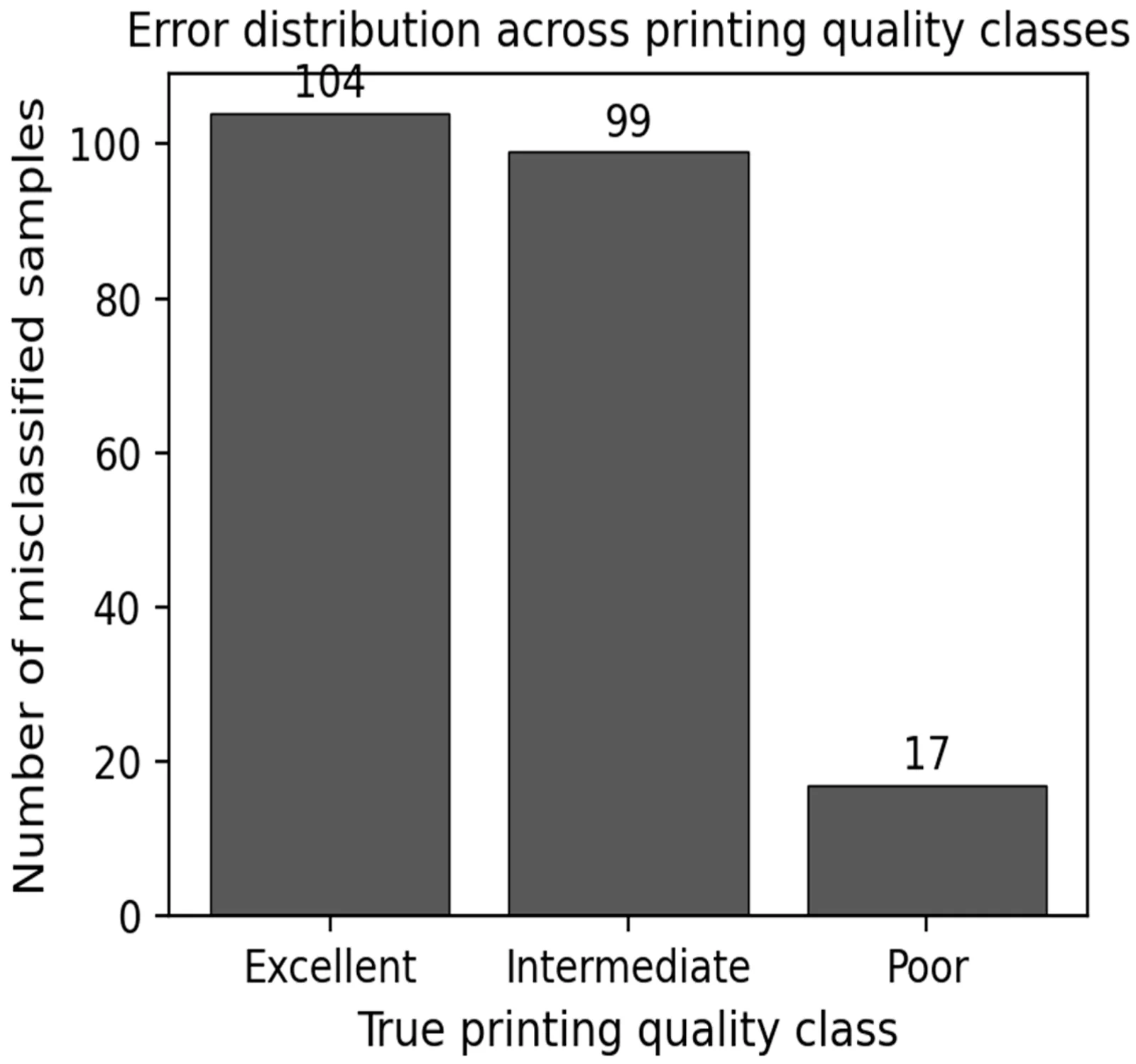
Misclassification histogram showing the number of misclassified samples per true printing-quality class. The Poor class has the fewest errors (n = 17), confirming how separable it is; Excellent and Intermediate are confused with each other more often (n = 104 and n = 99, respectively), which fits their gradual morphological transition.

**Figure 5:**
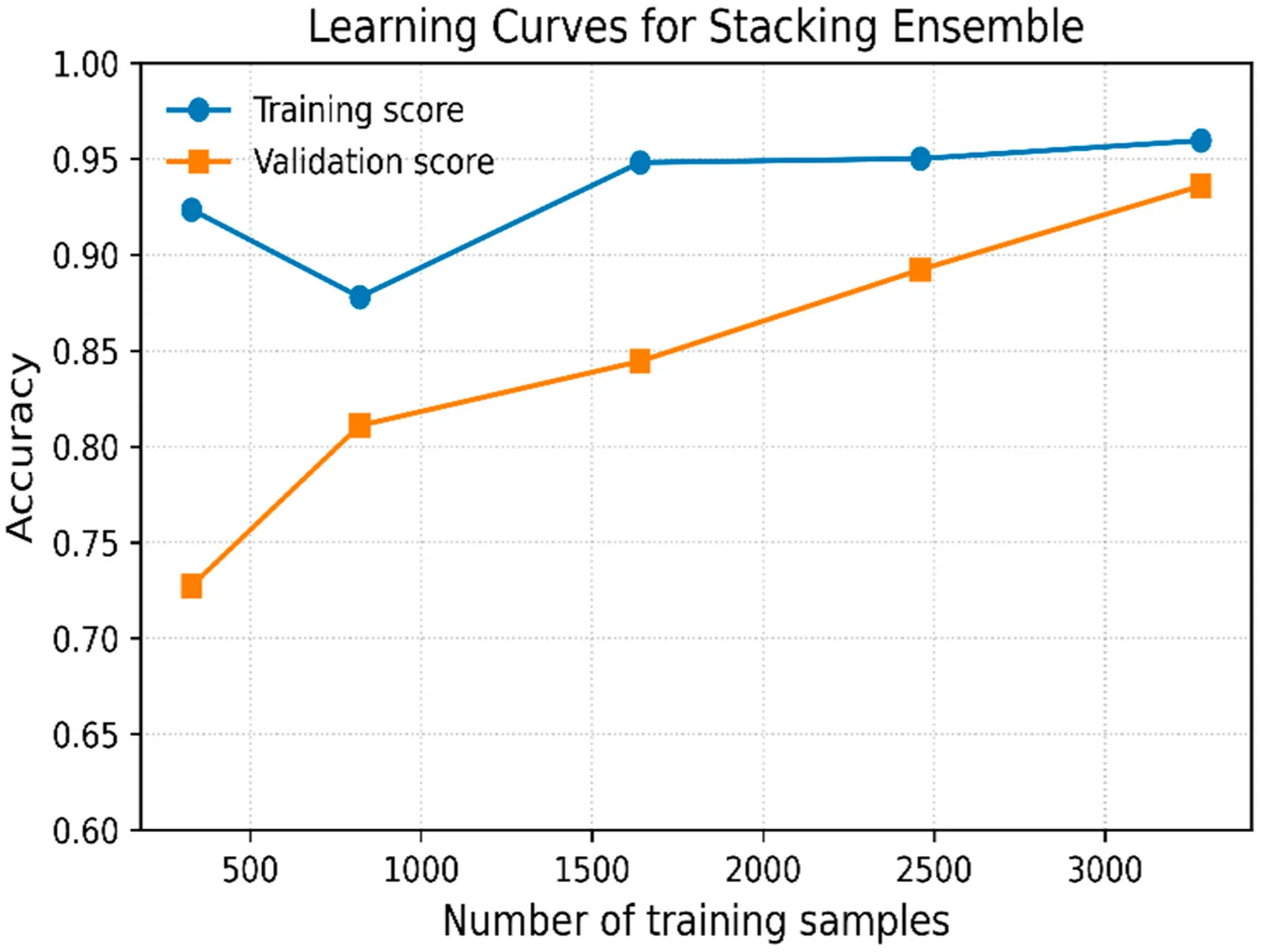
Learning curves for the stacking ensemble. Training and validation accuracy converge, with the validation score reaching roughly 0.94 at the full dataset size of 3280 samples; the small remaining gap points to no meaningful overfitting.

**Figure 6:**
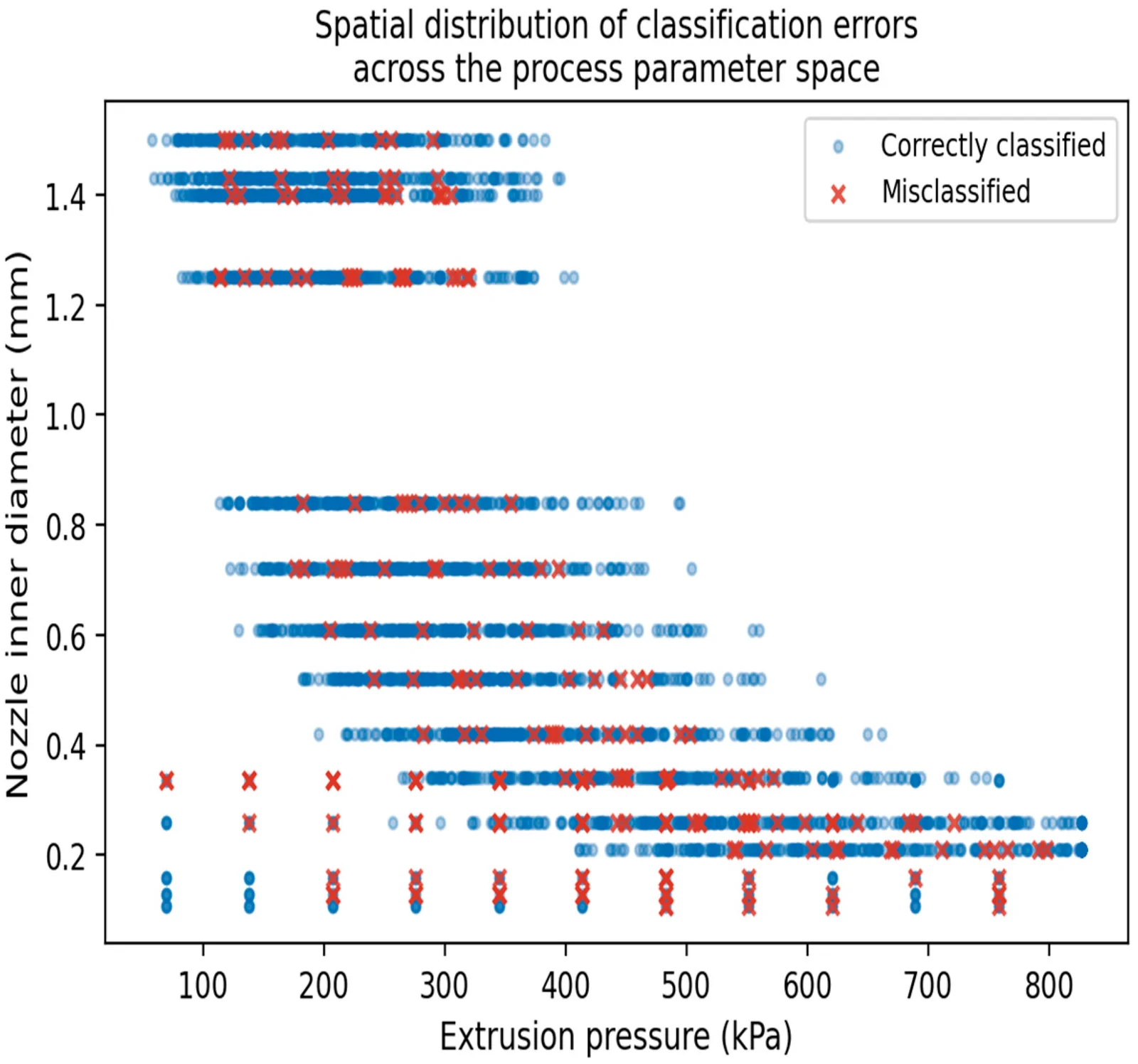
Spatial distribution of classification errors across extrusion pressure and nozzle inner diameter. Misclassifications concentrate at the finest nozzle diameters and mid-range pressures—the operating conditions where a small parameter change is most likely to push a sample across a class boundary.

**Figure 7:**
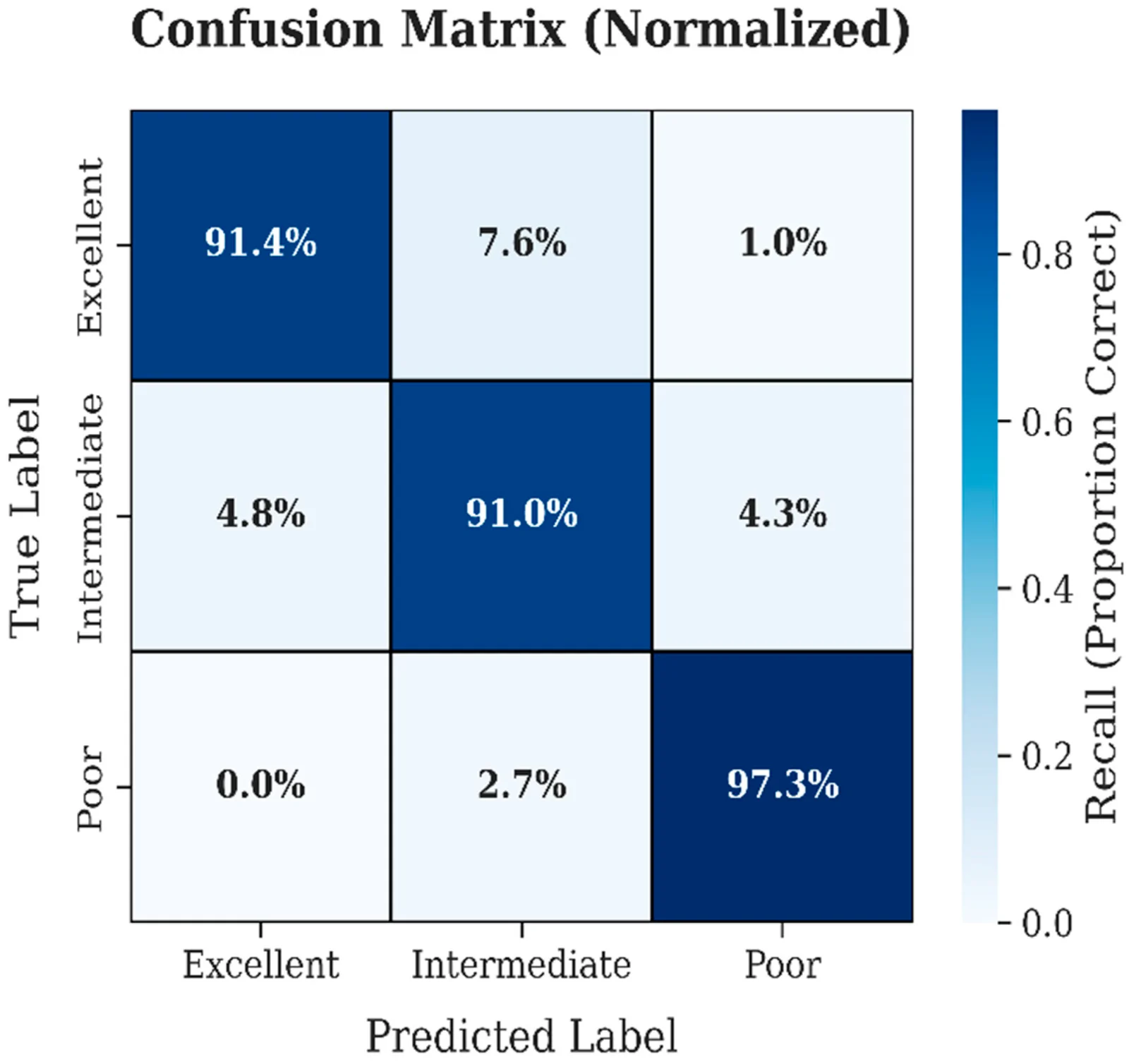
Normalized confusion matrix for the voting ensemble. Per-class recall: Excellent = 90.1%; Intermediate = 89.5%; Poor = 98.7%. Most errors fall at the Excellent–Intermediate boundary, which fits the smooth viscoelastic transition in Alg–MC gels.

Model behavior was examined with several complementary diagnostics, each reported in Section 4:

- One-vs-rest ROC curves (Figure 3) assessing class separability;
- A misclassification histogram (Figure 4) identifying uncertain parameter regions;
- Learning curves (Figure 5) tracking convergence with dataset size;
- An error-distribution plot (Figure 6) mapping where errors concentrate across the parameter space.
- A normalized confusion matrix (Figure 7) quantifying class-level misclassification;

Together, these diagnostics point to where the model can be trusted and where its predictions are least certain.

### 3.8. Ablation Experiments

**Figure 8:**
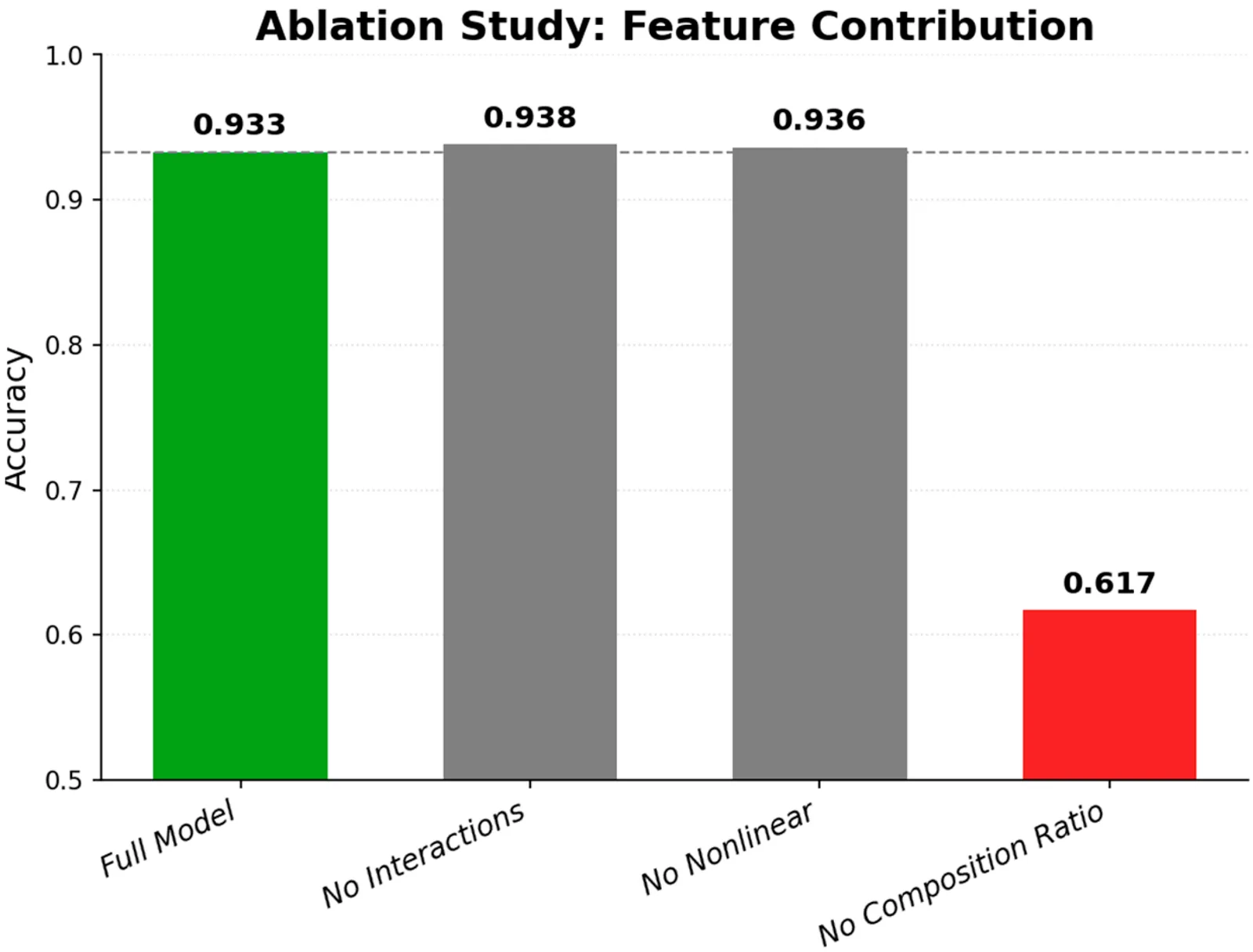
Ablation study. Removing the Alg:MC composition ratio reduces accuracy from 0.933 to 0.617 (ΔAcc = −0.316), whereas removing interaction terms or nonlinear transformations has a smaller effect, confirming that formulation chemistry is a major, though not sole, driver of printability prediction.

To quantify the contribution of each engineered feature group, composition-related features, pressure–geometry interaction terms, and nonlinear transformations were removed individually and the stacking ensemble retrained. As shown in Figure 8, removing the composition feature reduced accuracy sharply from 0.933 to 0.617 (ΔAcc = −0.316), far exceeding the effect of removing either other group or confirming that ink chemistry dominates extrusion behavior.

### 3.9. Reproducibility and Limitations

Analysis scripts and processed datasets supporting the findings of this study are contained within the article. The main methodological limitations are as follows: CFD validity here rests on mesh-independence convergence (Section 3.3) and on agreement between the fitted power-law viscosity parameters and published rheology for comparable Alg–MC formulations, rather than on a direct quantitative comparison against experimentally measured flow rate or filament geometry at matched conditions. That comparison is left as a priority for the extended validation study (Section 4.10).

- The CFD model excludes die swell, gravitational sag, and post-extrusion viscoelastic relaxation;
- DBN-generated samples may introduce residual distributional bias in sparsely sampled regions, despite reconstruction-error filtering;
- Printability labeling retains an element of expert judgment, although inter-rater agreement was strong (κ = 0.83);
- Validation used a single printer platform, so cross-platform generalization remains to be confirmed;
- Environmental factors such as temperature and humidity, which affect viscosity and crosslinking, were not modeled.

## 4. Results and Discussion

### 4.1. Dataset Characteristics

Before training, the hybrid dataset was checked to confirm that the experimental, CFD, and DBN-augmented samples formed a numerically consistent whole. Descriptive statistics for the two continuous inputs are given in Table 3.

Augmentation broadened the coverage of the input variables without exceeding the physical limits of the system, and the engineered features preserved consistent scaling across the three data sources, confirming the structural integrity of the combined dataset. Before fold-level stratification, the raw class distribution across the 3280 samples was 32.0% Excellent, 28.6% Intermediate, and 39.3% Poor—a systematic parameter sweep inevitably samples many combinations outside the printable window as well as within it. Stratification was applied only when constructing the cross-validation folds, so that each fold contained a representative share of all three classes; it did not rebalance the dataset’s underlying class distribution, which is the one reported above and used throughout evaluation.

### 4.2. Model Performance

Every classifier cleared 0.73 accuracy (Table 2), though not all reached 0.92—logistic regression, the weakest model, topped out at 0.733, well below the other six. The voting ensemble came out on top for accuracy and F1 (accuracy 0.933; F1 = 0.929), with the stacking ensemble statistically indistinguishable from it (accuracy 0.933; F1 = 0.928) and random forest close behind at 0.922. Across the five cross-validation folds, accuracy was 93.3% for the voting ensemble (95% CI: 92.5–94.1%) and 93.3% for the stacking ensemble (95% CI: 92.4–94.2%). A McNemar test on the paired out-of-fold predictions found no statistically significant difference between the two ensemble strategies (*p* = 1.00; 36 versus 35 discordant predictions out of 3280) so the voting ensemble’s slightly higher point estimate is not a meaningful advantage; both ensembles simply sit ahead of every individual base classifier. Gradient boosting and SVM landed in the middle, while logistic regression and k-nearest neighbors trailed, which fits a classification boundary between the Excellent and Intermediate classes that a linear or purely distance-based decision rule does not capture well. These results broadly agree with prior pharmaceutical-3DP findings that ensemble ML predicts printability more reliably than single models [19,20], and add to them by showing that a physics-informed hybrid dataset improves generalization.

### 4.3. Class-Level Behavior and Error Distribution

The normalized confusion matrix (Figure 7) shows most errors falling at the Excellent–Intermediate boundary—expected, since these two classes’ filament morphologies differ only gradually—while the Poor class stays clearly separated and is rarely misclassified.

The misclassification histogram (Figure 4) shows that uncertain predictions cluster near intermediate composition ratios and pressure-to-diameter values. These borderline conditions coincide with the region where Alg–MC inks transition between stable and unstable extrusion, in agreement with published rheological data showing a gradual viscosity change between the corresponding quality regimes.

### 4.4. ROC Analysis and Class Separability

The ROC curves (Figure 3) show strong separability: the Poor class reaches AUC = 0.998, followed by Excellent (0.990) and Intermediate (0.982). Because these AUCs stay high across the board, predictive uncertainty is concentrated at the Excellent–Intermediate boundary rather than spread across all classes—a favorable property for practical pre-print screening.

### 4.5. Learning Dynamics

The learning curves (Figure 5) show training and validation accuracy converging as the dataset grows; the validation score stabilizes near 0.94, and the training–validation gap stays below 0.02 at the full dataset size. That points to a well-fitted model and confirms that augmentation improved performance without introducing overfitting.

The spatial error map (Figure 6) shows misclassified points clustering where composition and pressure jointly produce borderline flow—mirroring the ablation results (Figure 8) and reinforcing how much a small composition change matters near the printability transition.

### 4.6. Contribution of Engineered Features

The ablation results in Figure 8 back up the feature-importance ranking from Figure 2—worth noting, since the two are measuring different things (Gini impurity reduction in one case, the accuracy cost of dropping a feature entirely in the other). Removing the interaction and nonlinear terms only lowered accuracy modestly; removing the composition feature, by contrast, dropped it from 0.933 to 0.617. Because the four pressure- and diameter-derived features (f_1_–f_4_) are deterministic algebraic transformations of P and d, they are necessarily correlated with the base inputs by construction. We do not report Pearson coefficients between them for this reason—the values would simply reflect algebra, not new empirical information. The ablation results are, in our view, a more informative test of the same question: once the composition feature is present, these four features carry comparatively little independent predictive value, which is the same redundancy a correlation analysis would only confirm indirectly. The tree-based and ensemble models used here (random forest, gradient boosting, and the stacking/voting ensembles) are also inherently robust to multicollinearity, since they select splits by information gain rather than requiring feature orthogonality the way linear models do, and each of the four terms is a single arithmetic operation, so their computational cost is negligible regardless of their marginal contribution. This fits polymer-network theory, in which the Alg:MC ratio governs the viscoelastic spectrum of the blend [21,36] and thereby sets the pressure–diameter window that yields stable filaments.

### 4.7. External Validation

On an independent hold-out set containing Alg:MC ratios and nozzle gauges absent from training, the voting ensemble reached 75.9% accuracy (precision 0.768, recall 0.724, and F1 0.729) on 871 samples drawn from a fully withheld composition ratio and needle gauge. Errors again concentrated at balanced composition ratios, where elastic and viscous contributions are comparable and small compositional changes have a large effect on the printed filament. The result supports generalizability while pinpointing parameter regions that would most benefit from additional experimental data.

### 4.8. Comparison with Conventional Approaches

Conventional optimization via Design of Experiments (DoE) can require hundreds of physical runs to map a multi-dimensional parameter space [10]. The present framework instead screens candidate conditions computationally before any material is printed, which is especially valuable when bioink or printer time is limited. Unlike single-variable printability indices such as the filament-collapse angle or the Pr-number [7,9], the model explicitly represents parameter interactions; the ablation experiments show that removing these interaction and composition features degrades accuracy, confirming their predictive value. This behavior is consistent with the rheological control that the Alg:MC ratio exerts over printability [36].

### 4.9. Practical Implications

#### 4.9.1. Rapid Formulation Screening

Ranking candidate conditions before printing lets the framework shorten formulation development from many iterative prints down to a focused set of confirmatory experiments—directly useful for the fast development of personalized, 3D-printed dosage forms.

#### 4.9.2. Quality Control and Training

Pre-print quality prediction lets operators reject unpromising parameter combinations in advance, cutting down failed prints and wasted material. It is currently a software tool only, but nothing rules out integrating it into printer control systems down the line. The same diagnostic plots are also useful for teaching, since they make the links between composition, machine settings, and print quality concrete for anyone new to extrusion bioprinting.

### 4.10. Limitations and Future Directions

The main limitations follow directly from the study design. The framework was developed and validated for Alg–MC inks, so extension to other hydrogels will require new experimental data. Environmental variables (temperature and humidity) and printer-to-printer differences were not modeled and would improve robustness if included. Biological outcomes such as cell viability and post-printing mechanical properties were outside the present scope; a complete decision-support system would treat printability as one objective within a multi-objective framework. Finally, the DBN-augmented data may introduce mild bias near sparsely sampled boundaries, so additional experiments at the Excellent–Intermediate transition would further strengthen the model. Future work will pursue quantitative image-based printability scoring, inclusion of environmental and printer descriptors, transfer learning to new inks from small datasets, and closed-loop control that adjusts pressure and speed in real time.

## 5. Conclusions

We developed and validated a physics-informed machine-learning framework that predicts the extrusion print quality of alginate–methylcellulose bioinks before printing. By combining experimental data, CFD nozzle-flow simulations, and DBN augmentation into a single consistent dataset of 3280 samples, and by engineering features grounded in extrusion physics, the voting ensemble reached 93.3% cross-validated accuracy and 75.9% accuracy on a genuinely withheld composition ratio and needle gauge, with per-class AUCs of 0.97–0.99. Feature-importance and ablation analyses identified the Alg:MC ratio, extrusion pressure, and nozzle diameter as the dominant factors, in agreement with established rheology. The framework predicts extrusion printability only; it does not validate downstream biological or pharmaceutical performance—cell viability, drug-release kinetics, and clinical dosage-form efficacy—which remain targets for future work. Within that scope, the framework offers a practical, interpretable route to reducing experimental burden in pharmaceutical bioink development, and lays groundwork for eventually integrating with real-time printer control—work that connects naturally to our group’s broader interest in AI-integrated pharmacy systems. The gap between the cross-validated accuracy and the external-validation accuracy deserves direct comment. Five-fold cross-validation shows how the model performs on data drawn from the same overall distribution it was trained on; on its own, that does not establish generalization to a formulation or nozzle geometry that the model has never seen in any form during training. The external-validation protocol here is deliberately stricter than a random held-out split—an entire composition ratio and an entire needle gauge were withheld from training in full—so the reported 75.9% reflects performance under genuine distributional shift rather than interpolation within a familiar parameter range. A gap of this kind between in-distribution and out-of-distribution performance is expected, and we think that reporting it plainly, rather than presenting only the more favorable in-distribution figure, gives a more honest picture of the model’s current practical reach [38] and in adaptive, AI-driven control architectures more generally [39].

## Figures and Tables

**Table 1:** Input parameters and operational ranges.

Parameter	Range or Value	Unit	Notes
Alg:MC ratio	1:1, 1:1.5, 1:2, 1:2.5, 1:3	weight ratio	Set by dry mass
Needle gauge	14–32	gauge	Standard blunt-tip sets
Inner diameter	0.108–1.50	mm	Based on gauge chart
Extrusion pressure	57.2–827.4	kPa	8.3–120 psi at printer inlet
Print quality	Excellent, Intermediate, Poor	class	Target label

Abbreviation: kPa, kilopascal (1 psi = 6.895 kPa).

**Table 2:** Performance metrics for all classification models (five-fold stratified cross-validation, n = 3280).

Model	Accuracy	Precision	Recall	F1-Score
Stacking Ensemble	0.933	0.929	0.928	0.928
Voting Ensemble	0.933	0.931	0.927	0.929
Random Forest	0.922	0.917	0.915	0.916
Gradient Boosting	0.910	0.906	0.904	0.905
SVM	0.923	0.922	0.918	0.919
Logistic Regression	0.733	0.714	0.716	0.713
K-Nearest Neighbors	0.919	0.916	0.914	0.914

Metrics are macro-averaged over the three printability classes. The voting and stacking ensembles achieved statistically indistinguishable performance (McNemar test on paired out-of-fold predictions, *p* = 1.00, 36 versus 35 discordant predictions out of 3280); both ensembles perform reliably ahead of every individual base classifier. SVM, support vector machine; KNN, k-nearest neighbors.

**Table 3:** Descriptive statistics of the final hybrid dataset (n = 3280).

Variable	Count	Mean	SD	Min	25%	Median	75%	Max
Inner diameter (mm)	3280	0.752	0.471	0.108	0.340	0.610	1.250	1.500
Extrusion pressure (kPa)	3280	341.7	179.6	57.2	206.8	299.2	444.8	827.4

kPa values converted from psi (1 psi = 6.895 kPa). SD, standard deviation.

## Data Availability

The authors confirm that the data supporting the findings of this study are available within the article.
